# Integrative therapies for chronic insomnia: A randomized controlled trial of a traditional Thai Herbal Remedy and *Cannabis sativa* oil

**DOI:** 10.1016/j.sleepx.2026.100173

**Published:** 2026-01-17

**Authors:** Naruwat Pakdee, Nitcha Sribunrieng, Ronnachai Poowanna

**Affiliations:** Department of Thai Traditional Medicine, Faculty of Natural Resources, Rajamangala University of Technology Isan Sakon Nakhon Campus, 47160, Thailand

**Keywords:** Chronic insomnia, Integrative medicine, Thai traditional medicine, Herbal therapy, *Cannabis sativa*, Sleep quality

## Abstract

**Background:**

This study compared the efficacy and safety of integrative and conventional therapies for chronic insomnia.

**Objective:**

To evaluate the effects of the Suk-Sai-Yat traditional Thai herbal remedy, *Cannabis sativa* oil (Deja formula) and lorazepam on sleep quality and quality of life in patients with chronic insomnia.

**Methods:**

In a randomized controlled parallel-group trial, 60 adults with chronic insomnia received Suk-Sai-Yat, *Cannabis sativa* oil, or lorazepam for four weeks. Sleep quality was assessed using the Pittsburgh leep Quality Index (PSQI) and quality of life was evaluated using EQ-5D-5L and EQ-VAS. Safety was monitored throughout the study.

**Results:**

After four weeks, PSQI scores significantly improved in all groups: Suk-Sai-Yat (12.3–6.6), *Cannabis sativa* oil (13.6–3.68) and lorazepam (14.4–5.8) (all p < 0.001), with no significant differences between groups. Quality-of-life scores improved significantly in the integrative therapy groups. Only mild adverse events were reported.

**Conclusion:**

Suk-Sai-Yat and *Cannabis sativa* oil demonstrated comparable efficacy to lorazepam with favorable safety profiles, supporting their role as integrative, non-benzodiazepine options for chronic insomnia management.

## Highlights

• Suk-Sai-Yat and *Cannabis sativa* oil significantly improved chronic insomnia symptoms
• Herbal and cannabis treatments showed efficacy comparable to lorazepam in an RCT
• Quality of life improved more with Suk-Sai-Yat and cannabis oil than lorazepam
• Only mild adverse events were observed across all treatment groups
• Supports traditional herbal and cannabinoid therapies as safe insomnia alternatives

## Graphical abstract

**Figure:**
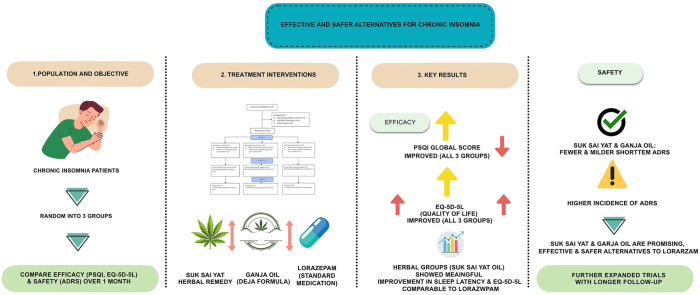

## 1 Introduction

Insomnia is a pervasive sleep disorder characterized by persistent difficulties in initiating or maintaining sleep or experiencing nonrestorative sleep despite adequate opportunity, leading to daytime impairment and reduced quality of life [1,2]. Globally, approximately 10–30 % of adults report chronic insomnia symptoms, with even higher prevalence in older populations and those with comorbid conditions [3,4]. In Thailand and globally, insomnia and poor sleep quality have been increasingly recognized in community- and hospital-based surveys, contributing significantly to physical and mental health burdens [5]. Pharmacological treatments using benzodiazepines, such as lorazepam, are standard for managing moderate to severe insomnia and anxiety-associated sleep disturbances owing to their efficacy and rapid onset of action [6,7]. However, these agents carry potential risks such as tolerance, dependence, residual daytime sedation, cognitive impairment and rebound insomnia upon discontinuation [8,9]. The side effects and risk profile often limit the long-term utility of such medications and drive interest in safer alternatives [10].

Herbal and alternative therapies have been explored as potential treatments for insomnia, with lower adverse effects. A systematic review by Ni et al. [11,12] found that Chinese herbal medicines significantly improved subjective sleep quality, latency, and duration in randomized controlled trials. Similarly, Yang et al. [13] reported that combining herbal decoctions with lorazepam enhanced its effects while reducing benzodiazepine-related side effects. Other herbal interventions such as Hwanglyeonhaedok-tang have also shown efficacy in improving sleep parameters in clinical trials [14,15].

In Thailand, Suk-Sai-Yat, a cannabis-containing traditional remedy, was found to significantly reduce Pittsburgh Sleep Quality Index (PSQI) global scores in patients with chronic insomnia in follow-ups of several months [5]. The Suk-Sai-Yat remedy comprises 12 medicinal botanicals formulated according to classical Thai traditional medicine. The formulations included *Cannabis sativa* L. (*C. sativa*) (12 g), *Piper retrofractum* Vahl (11 g), *Zingiber officinale* Roscoe (10 g), *Piper nigrum* L. (9 g), *Mesua ferrea* L. (8 g), *Myristica fragrans* Houtt. (7 g); *Aucklandia lappa* Decne. (6 g), *Nigella sativa* L. (5 g), *Cinnamomum burmannii* (Nees & T. Nees) Blume (4 g), *Clausena excavata* Burm. (3 g) and *Azadirachta indica* A. Juss. var. *siamensis* Valeton (2 g) and *Cinnamomum camphora* (L.) J. Presl (1 g).

Several key constituents such as *C.* *sativa*, *Piper retrofractum*, *Zingiber officinale*, *Piper nigrum*, and *Azadirachta indica* have been traditionally used to promote sedation, reduce anxiety and restore “Lom” (wind element) balance, a fundamental pathological concept in Thai traditional medicine associated with sleep regulation and autonomic equilibrium. Modern pharmacological evidence supports these traditional uses: cannabidiol from piperine-rich species including *Piper retrofractum* and *Piper nigrum* demonstrate anxiolytic and GABAergic activity [17], and *Zingiber officinale* extracts show central nervous system depressant and anxiolytic effects [18]. Extracts of *Azadirachta indica* have also exhibited anxiolytic and antidepressant properties in preclinical models [19], supporting their traditional use in formulations aimed at regulating sleep and autonomic balance. The ingredients in the Suk-Sai-Yat remedy have pharmacologically validated sedative and anxiolytic properties. For example, *C.* *sativa* leaves contain cannabidiol, which exerts sedative and hypnotic effects through 5-HT1A receptor activation [16]. *Piper retrofractum* and *Piper nigrum* fruits contain piperine and monoterpenes with anxiolytic and anticonvulsant activities. *Myristica fragrans* enhances both light and deep sleep, whereas *Zingiber officinale* exhibits anxiolytic and neuroprotective actions [20]. Other components, such as *Cinnamomum camphora* (borneol) and *Mesua ferrea* (β-sitosterol), also demonstrate sedative or anti-inflammatory effects [21]. These combined pharmacological actions support the traditional use of Suk-Sai-Yat for sleep induction and justify its use in controlled clinical settings. A retrospective comparative study in Phunphin Hospital compared herbal formulas, including Ya Suk-Sai-yat, Yahom Thephachit, and cannabis oil (Deja formula) and found no significant differences in reducing insomnia severity [22]. Moreover, Herbal extracts have been shown to possess melatonin or melatonin-like activity, which may underlie some of their sleep-modulating effects [23,24].

Recent trials on cannabinoids for sleep have produced promising results. For example, a pilot randomized controlled trial of 150 mg cannabidiol (CBD) nightly in individuals with primary insomnia reported improvements in objective sleep efficiency and subjective well-being relative to placebo [25]. Another study investigating non-psychoactive cannabinoids reported longer sleep durations in the CBD group than in the placebo [26]. Additionally, formulations combining CBD with terpenes or other cannabinoids (such as cannabinol, CBN) have been examined in randomized, double-blind, placebo-controlled designs, showing reductions in awakening and overall sleep disturbance, although not always in sleep-onset latency [27,28]. From an ethnobotanical perspective, other herbal species, such as *Valeriana officinalis*, *Matricaria chamomilla* (chamomile), *Viola odorata*, hops (*Humulus lupulus*) and *Piper nigrum* (pepper leaves), have been evaluated. A recent pilot trial in Hat Yai, Thailand, comparing pepper leaf tea with chamomile tea, found that both significantly decreased insomnia severity (Insomnia Severity Index, ISI), improved PSQI sleep quality and reduced stress, as measured by ST-5 [29]. In global herbal supplement reviews, valerian, melatonin, chamomile, hops, kava and tryptophan are among those with the most consistent, although moderate, evidence [30,31].

Despite this growing body of evidence, direct head-to-head comparisons between traditional remedies (particularly those containing cannabis and herbal combinations) and standard pharmacotherapy, such as lorazepam, in a controlled randomized design remain limited. There is a gap in rigorous clinical trials that not only assess sleep quality via validated instruments (e.g., PSQI), but also measure health-related quality of life, adverse reactions, and compare multiple treatment arms including herbal, cannabis-based, and pharmacologic agents within the same cohort study. Current clinical guidelines increasingly recommend non-benzodiazepine approaches as first-line treatments for chronic insomnia, including cognitive behavioral therapy for insomnia (CBT-I), non-benzodiazepine hypnotics and selected integrative therapies, due to concerns regarding tolerance, dependence, and adverse cognitive effects associated with long-term benzodiazepine use [1,32]. Traditional herbal remedies and cannabis derived preparations have increasingly gained attention as potential complementary or alternative approaches; however, evidence regarding their long term safety remains limited [33,34].

The present study aimed to directly compare the effectiveness and safety of Suk-Sai-Yat herbal remedy, *C.* *sativa* oil or Ganja oil (Deja formula) and lorazepam in patients with chronic insomnia over a one-month period. The primary outcome was sleep quality (PSQI); secondary outcomes included health-related quality of life (EQ-5D-5L) and the incidence and severity of adverse drug reactions.

## 2 Method

### 2.1 Study design

**Fig. 1:**
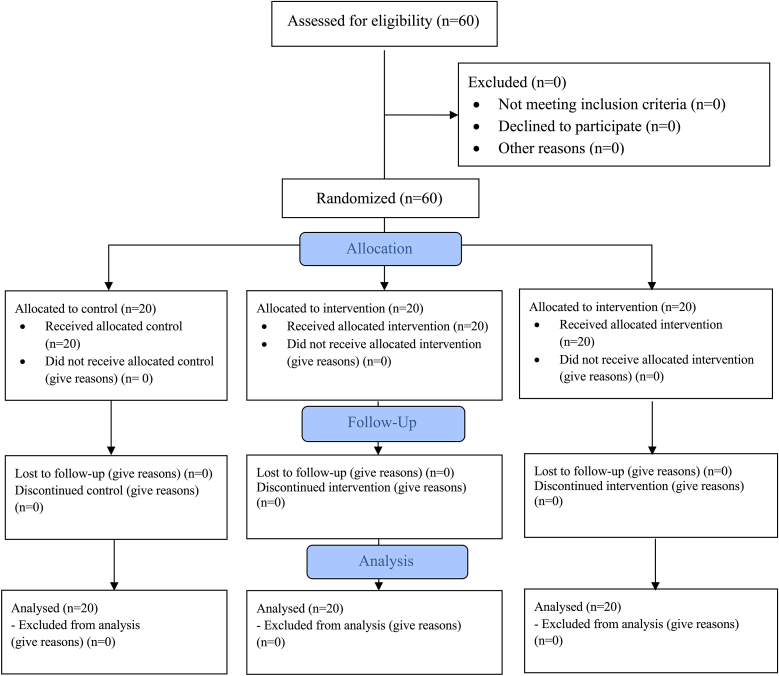
CONSORT Flow diagram of participant recruitment, randomization, follow-up and analysis.

This study was a randomized controlled parallel-group clinical trial designed to compare the efficacy and safety of three therapeutic interventions for chronic insomnia: Suk-Sai-Yat (Traditional Thai Herbal Remedy), *C.* *sativa* oil (Deja formula) and lorazepam (1 mg tablet). The trial followed the consolidated standards of reporting trials (CONSORT shown in Fig. 1) recommendations for parallel-group clinical studies and was conducted over a 4-week intervention period with assessments at baseline (week 0 and post treatment (week 4). This study adhered to the principles of the Declaration of Helsinki and Good Clinical Practice (GCP). The trial was implemented at Sai Ngam Hospital, a regional healthcare facility with a dedicated Thai traditional medicine unit and outpatient department specializing in chronic diseases and complementary medicine services.

### 2.2 Participants

The trial was conducted at the outpatient department of Sai Ngam Hospital, a secondary care public hospital serving rural and semi-urban populations in Kamphaeng Phet Province, Thailand. A total of 92 patients were initially screened between June and August 2023. Of these, 60 met the eligibility criteria and were enrolled in the study. The screening included medical history, physical examination, and completion of the Pittsburgh Sleep Quality Index (PSQI). Participants younger than 40 years were excluded to reduce clinical heterogeneity associated with transient, stress-related, or circadian rhythm–related insomnia, which has been reported to be more prevalent in younger adults. In contrast, middle-aged and older adults are more likely to experience chronic insomnia with relatively stable symptom patterns, allowing for a more homogeneous clinical sample and improved internal validity [1].

#### 2.2.1 Inclusion criteria

Diagnosis of chronic insomnia was defined by the DSM-5criteria [2], age between 40 and 70 years, Global PSQI score ≥5 [35] and the ability to provide informed consent and comply with follow-up visits.

#### 2.2.2 Exclusion criteria

Current use of hypnotics, antidepressants, or sedative medications; severe psychiatric or neurological disorders (e.g., schizophrenia, major depression, epilepsy); pregnancy or breastfeeding; and known hypersensitivity to herbal products or cannabis derivatives.

### 2.3 Sample size and randomization

The sample size was calculated to detect a medium effect size (Cohen's d = 0.5) in PSQI score differences, with 80 % power and a two-tailed alpha of 0.05, resulting in a minimum of 18 participants per group. To compensate for a potential 10 % dropout rate, 20 participants were recruited per group, yielding a total sample size of 60 participants. Randomization was performed using a computer-generated sequence with block randomization (block size = 6) to ensure equal allocation among the three study arms. Allocation concealment was achieved using sequentially numbered, opaque, sealed envelopes. The allocation sequence was concealed until the time of assignment, and personnel involved in participant enrollment and intervention assignment did not have access to the randomization sequence, thereby minimizing selection bias.

Due to the distinct characteristics and routes of administration of the interventions, blinding of participants and care providers was not feasible. However, outcome assessors and data analysts were blinded to group allocation using anonymized group codes, and group assignments were disclosed only after completion of data analysis.

### 2.4 Interventions

**Group 1** Herbal remedy: Suk-Sai-Yat herbal remedy in capsule form (500 mg/capsule) administered orally twice daily after meals for 4 weeks. The formulation consisted of 12 medicinal plants in proportional ratios (1–12 parts). Each component has documented phytochemical and biological activities relevant to sleep induction, anxiolysis, or autonomic regulation. Key constituents include cannabidiol from *C.* *sativa*, piperine from *Piper retrofractum*, gingerols and shogaols from *Zingiber officinale* [36]. Fresh ginger's 10-gingerol further demonstrates the capacity to suppress neuroinflammation by blocking NF-κB activation and lowering nitric oxide, IL-6 and TNF-α levels [37]. trimyristin from *Myristica fragrans* and borneol from *Cinnamomum camphora.* These compounds collectively provide sedative, anxiolytic, neuroprotective, anti-inflammatory and autonomic-modulating actions, forming the traditional rationale for their use in treating insomnia.
**Group 2** *Cannabis sativa* oil: *C.* *sativa* oil (Deja formula) provided by the Thai traditional medicine unit, administered subcutaneously at a dose of one drop (0.05 mL) nightly before bedtime.
**Group 3** Reference pharmacological comparator: Lorazepam 1 mg tablet administered orally nightly before bedtime. Adherence was monitored using pill counts, patient diaries, and weekly telephone check-ins. Lorazepam was selected as the pharmacological comparator because it remains commonly prescribed for short term insomnia management in routine clinical practice, particularly in hospital and primary care settings. Although non-benzodiazepine agents are increasingly recommended, benzodiazepines continue to be used due to availability, clinician familiarity, and cost considerations. The 1 mg nightly dose was chosen based on standard prescribing guidelines (0.5–2 mg), representing a conservative and commonly used starting dose that balances hypnotic efficacy and safety. Lorazepam served as a clinically relevant benchmark rather than a pharmacological equivalent to the integrative interventions [[38], [39], [40]].

Treatment adherence was monitored through pill counts, patient diaries, and weekly telephone follow-ups conducted by clinical staff. These measures were implemented to promote compliance and verify adherence throughout the intervention period [41]. Adherence across all intervention groups was monitored using pill counts, patient diaries and weekly telephone follow ups conducted by clinical staff.

### 2.5 Outcome measures

Primary outcome: Sleep quality was assessed using the Pittsburgh Sleep Quality Index (PSQI) [35,42] and evaluated in seven domains: subjective sleep quality, sleep latency, sleep duration, habitual sleep efficiency, sleep disturbances, use of sleep medication and daytime dysfunction.

Secondary outcomes: Health-related quality of life was measured using the EQ-5D-5L instrument and the EQ-VAS scale [43,44] and adverse drug reactions were evaluated through structured questionnaires and open reporting at each visit. The classification follows the WHO-UMC causality assessment system [45]. The severity was graded as mild, moderate, or severe.

### 2.6 Data collection procedure

Baseline data collection included sociodemographic characteristics (age, sex, marital status, education, and occupation) and clinical characteristics (duration of insomnia and comorbidities). Follow-up assessments were performed after four weeks. All assessments were conducted face-to-face by trained research nurses, who were blinded to the intervention groups. The research nurses conducted face-to-face interviews during each visit. Clinical pharmacists monitored medication adherence and ADRs. Missing data were minimized by follow-up phone calls and appointment reminders. No interim analyses were planned or conducted. The study was carried out according to a predefined protocol with a fixed sample size and study duration. Stopping criteria were predefined for safety reasons, and the trial would be discontinued if participants experienced severe allergic reactions, serious adverse events, or any conditions posing unacceptable risk to participant safety.

### 2.7 Statistical analysis

Descriptive statistics (mean, standard deviation, frequency and percentage) were computed for baseline characteristics. Within-group comparisons of pre- and post-intervention scores were performed using paired t tests. Between-group comparisons were performed using one-way ANOVA. Post-hoc comparisons were performed using Bonferroni correction. Normality was verified using the Shapiro-Wilk test. Non-parametric tests (Kruskal-Wallis) were applied when the assumptions were violated. Statistical significance was set at P < 0.05. Statistical analyses were conducted using SPSS version 26.0 (IBM Corp., Armonk, NY, USA).

## 3 Results

### 3.1 Participant characteristics

Sixty patients with chronic insomnia were enrolled and randomly divided into three groups: Suk-Sai-Yat herbal remedy (n = 20), *C.* *sativa* oil (Deja formula) (n = 20), and lorazepam (n = 20). The baseline demographic characteristics, including age and sex distribution, were comparable among the three groups, with no statistically significant differences (p > 0.05) (Table 1).

**Table 1:** Baseline demographic and clinical characteristics of participants.

Characteristic	Suk-Sai-Yat (n = 20)	*Cannabis sativa* oil (n = 20)	Lorazepam (n = 20)	p-value
Age, (mean ± SD) (years)	52.3 ± 9.8	54.1 ± 10.5	53.6 ± 8.9	0.72
Sex, n (%)				
• Male	8 (40 %)	7 (35 %)	9 (45 %)	0.88
• Female	12 (60 %)	13 (65 %)	11 (55 %)	0.79
Duration of insomnia, (mean ± SD) (years)	3.2 ± 1.4	3.5 ± 1.7	3.4 ± 1.6	0.81
Baseline PSQI, (mean ± SD)	12.25 ± 3.99	13.61 ± 3.68	14.35 ± 3.93	0.18
Baseline EQ-5D-5L, (mean ± SD)	0.90 ± 0.06	0.79 ± 0.12	0.81 ± 0.16	0.03
Baseline EQ-VAS, (mean ± SD)	57.22 ± 11.14	46.50 ± 10.40	50.80 ± 13.73	0.03

Values are presented as mean ± SD or n (%). Between-group comparisons used one-way ANOVA for continuous variables and Chi-square test for categorical variables.

### 3.2 Sleep quality (PSQI)

At baseline, mean PSQI scores were 12.25 ± 3.99 in the Suk-Sai-Yat group, 13.61 ± 3.68 in the *C. sativa* oil group and 14.35 ± 3.93 in the lorazepam group. After 4 weeks of intervention, significant improvements were observed within all three groups: PSQI decreased to 6.60 ± 3.66 (p = 0.001), 5.10 ± 1.77 (p = 0.001), and 5.80 ± 2.95 (p = 0.001), respectively. Between-group comparisons using one-way ANOVA revealed no statistically significant differences in PSQI scores either at baseline (p = 0.18) or post-treatment (p = 0.27), indicating the comparable efficacy of the three interventions (Table 2). In addition to improvements in PSQI global scores, domain-level analysis demonstrated reductions in sleep latency, improvements in sleep duration and habitual sleep efficiency, and decreased daytime dysfunction across all intervention groups. No statistically significant between-group differences were observed for any PSQI subcomponent following the 4-week intervention (Supplementary Table S1).

**Table 2:** Pittsburgh Sleep Quality Index (PSQI) global scores before and after the 4-week intervention.

Group	Baseline (Mean ± SD)	Post-treatment (Mean ± SD)	p-value
Suk-Sai-Yat remedy	12.25 ± 3.99	6.60 ± 3.66	<0.001
*C. sativa* oil (Deja formula)	13.61 ± 3.68	5.10 ± 1.77	<0.001
Lorazepam	14.35 ± 3.93	5.80 ± 2.95	<0.001

### 3.3 Health-related quality of life (EQ-5D-5L index)

Baseline EQ-5D-5L index scores were 0.90 ± 0.06 in the Suk-Sai-Yat group, 0.79 ± 0.12 in the *C. sativa* oil group and 0.81 ± 0.16 in the lorazepam group. After treatment, values increased significantly within groups to 0.94 ± 0.09 (p = 0.02), 0.95 ± 0.03 (p = 0.01) and 0.86 ± 0.15 (p = 0.001), respectively. Between-group analysis showed a statistically significant difference both at baseline (p = 0.03) and post-treatment (p = 0.02), indicating that improvements in the herbal and cannabis groups were greater than those in the lorazepam group (Table 3).

**Table 3:** EQ-5D-5L index scores before and after treatment.

Group	Baseline (Mean ± SD)	Post-treatment (Mean ± SD)	p-value
Suk-Sai-Yat remedy	0.90 ± 0.06	0.94 ± 0.09	0.02
*C. sativa* oil (Deja formula)	0.79 ± 0.12	0.95 ± 0.03	0.01
Lorazepam	0.81 ± 0.16	0.86 ± 0.15	<0.001

### 3.4 Visual analogue scale (EQ-VAS)

Mean EQ-VAS scores at baseline were 57.22 ± 11.14 in the Suk-Sai-Yat group, 46.50 ± 10.40 in the *C. sativa* oil group and 50.80 ± 13.73 in the lorazepam group. After treatment, scores improved significantly to 84.17 ± 9.89 (p = 0.001), 82.25 ± 10.70 (p = 0.001) and 59.20 ± 8.62 (p = 0.01), respectively. Between group analysis showed significant differences both at baseline (p = 0.03) and after the intervention (p = 0.02), with the herbal and cannabis groups achieving substantially greater improvements than lorazepam (Table 4).

**Table 4:** EQ-VAS scores before and after treatment.

Group	Baseline (Mean ± SD)	Post-treatment (Mean ± SD)	p-value
Suk-Sai-Yat remedy	57.22 ± 11.14	84.17 ± 9.89	<0.001
*C. sativa* oil (Deja formula)	46.50 ± 10.40	82.25 ± 10.70	<0.001
Lorazepam	50.80 ± 13.73	59.20 ± 8.62	0.01

### 3.5 Adverse drug reactions

Adverse drug reactions were mild and self-limiting across all the groups. In the Suk-Sai-Yat group, two patients (10 %) reported palpitations and muscle pain. In the *C. sativa* oil group, headache, dizziness, and nausea were reported in 1 patient (5 %). In the lorazepam group, no serious adverse events were recorded, although mild daytime drowsiness was noted in some participants. No severe or life-threatening ADRs occurred in any of the groups (Table 5).

**Table 5:** Incidence and type of adverse drug reactions.

Group	Incidence (%)	Type of ADR	Severity
Suk-Sai-Yat remedy	10 % (2/20)	palpitations, muscle pain	mild
*C. sativa* oil (Deja formula)	5 % (1/20)	headache, dizziness, nausea	mild
Lorazepam	0 %	none reported	No ADRs

All three interventions significantly improved sleep and health-related quality of life in patients with chronic insomnia. Improvements in PSQI outcomes were statistically significant within groups, but not between groups. For quality of life measures (EQ-5D-5L and EQ-VAS), greater gains were observed in the herbal and cannabis groups than in the lorazepam group. Adverse events were rare, mild and manageable, indicating favorable safety profiles for both Suk-Sai-Yat and Ganja oil.

## 4 Discussion

Chronic insomnia, the most common sleep disorder, is a frequent problem in many people worldwide. It is characterized by difficulties in initiating sleep and maintaining sleep continuity. If left untreated, it can adversely affect overall health, quality of life and cognition. This randomized controlled trial compared the effects of Suk-Sai-Yat (Traditional Thai Herbal Remedy), *C. sativa* oil (Deja formula), and lorazepam on sleep and health-related quality of life among adults with chronic insomnia. All three interventions produced significant improvements in PSQI global scores and EQ-5D-5L index, indicating that each modality offers measurable clinical benefits. Notably, the magnitude of improvement among the herbal and cannabis groups was comparable to that of lorazepam, with a trend toward a better self-rated quality of life. The herbal formulations demonstrated only mild and self-limiting adverse reactions, whereas no ADRs were reported in the lorazepam group (Table 5). Although this pattern did not indicate superior safety for herbal interventions, the overall tolerability of all treatments was acceptable. This finding is consistent with prior literature noting the long-term risk–benefit limitations associated with benzodiazepines, despite their short-term tolerability [8,9].

### 4.1 Improvements in sleep quality (PSQI)

All three interventions significantly improved subjective sleep quality as measured by the Pittsburgh Sleep Quality Index (PSQI). The reduction in PSQI scores observed in the Suk-Sai-Yat (from 12.25 to 6.60), *C. sativa* oil (13.61–5.10) and lorazepam groups (14.35–5.80) aligns with global evidence that both herbal and conventional pharmacological treatments can reduce insomnia severity [35,42]. The magnitude of improvement in this study is consistent with meta-analyses of herbal interventions such as *Valeriana officinalis*, *Matricaria chamomilla* and *Suanzaoren decoctions*, which demonstrated clinically meaningful decreases in PSQI global scores [[10], [11], [12]]. Consistent with the global PSQI findings, analysis of individual PSQI subcomponents demonstrated comparable improvements across all treatment groups, with no statistically significant between-group differences observed for subjective sleep quality, sleep latency, sleep duration, or daytime dysfunction (Supplementary Table S1). These findings support the interpretation that the observed clinical benefits were broadly distributed across multiple dimensions of sleep quality rather than driven by a single domain. Prior randomized controlled trials combining Chinese herbal formulations with lorazepam have reported additive or synergistic effects with a lower incidence of adverse events [11,12], while systematic reviews highlight that benzodiazepines reliably reduce sleep latency but carry higher risks of tolerance and dependence [1,7,8]. Cannabis derived formulations are of particular interest given the growing body of evidence linking cannabinoids to sleep regulation. Supporting this emerging evidence, a prospective controlled clinical study conducted in Thailand reported significant improvements in subjective sleep quality and quality of life following three months of cannabis oil administration, without serious adverse events [46]. Cannabinoid receptor agonism, particularly via CB1, has been shown to influence slow-wave sleep and sleep continuity [[47], [48], [49]]. Pilot clinical trials of cannabidiol (CBD) have reported improved subjective sleep efficiency [25] and reduced awakenings [27] supporting the efficacy observed in our *C. sativa* oil group.

From a mechanistic perspective, Suk-Sai-Yat comprises multiple botanicals traditionally used to alleviate ‘wind element’ (Lom) imbalance in Thai traditional medicine. Experimental and pharmacological studies suggest that these constituents may exert sedative and anxiolytic effects through modulation of GABAergic pathways, anti-inflammatory activity, and autonomic regulation, processes that have been implicated in insomnia pathophysiology [50,51]. Autonomic balancing, supporting parasympathetic activation before sleep. Many herbal formulas in traditional systems exhibit synergistic effects, where multi-constituent interactions produce more balanced sedation than single-agent hypnotics. Cannabinoids act primarily through: CB1 receptor activation, reducing neuronal excitability. 5-HT1A receptor modulation, contributing to anxiolysis. Endocannabinoid system regulation, which plays a key role in circadian rhythm and sleep–wake cycles [47]. Recent clinical trials have reported that cannabis oils can extend the total sleep time and reduce sleep disturbances with favorable tolerability [27]. The present findings support these mechanisms by demonstrating significant improvements in the subjective sleep metrics.

Although no statistically significant between-group differences were observed, improvements in PSQI global scores exceeding 3 points are generally considered clinically meaningful [35]. Most participants across all groups achieved this threshold. The herbal and cannabis-based interventions demonstrated short-term improvements comparable to lorazepam, with adverse events that were predominantly mild and self-limiting during the study period.

Given the global push toward reducing benzodiazepine prescriptions due to safety concerns, the availability of validated herbal and cannabis-based alternatives is of high clinical value, especially in resource-limited or culturally traditional settings, including many regions of Africa and Asia. Additionally, herbal medicines are typically more accessible and affordable, making them suitable for primary care and community based treatment programs [52,53]. Nevertheless, interpretation of these findings should be made cautiously. A major limitation of this study is the reliance on subjective sleep assessments without objective measures such as polysomnography or actigraphy. Although validated instruments such as the PSQI are widely used in insomnia research, the absence of objective sleep data limits conclusions regarding sleep architecture and may affect the generalizability of the findings [35,54].

### 4.2 Improvements in health-related quality of life (EQ-5D-5L, EQ-VAS)

Significant improvements in EQ-5D-5L index values and EQ-VAS scores in the Suk-Sai-Yat and *C. sativa* oil groups reflected not only better sleep but also broader benefits in physical and psychological well-being. Insomnia is strongly associated with impaired quality of life, reduced work productivity and increased healthcare utilization [55,56]. Improvements in sleep often lead to parallel gains in mobility, self-care, mood and social functioning, as captured in EQ-5D-5L measures [44]. The particularly large improvement in EQ-VAS scores in the herbal and cannabis groups (from ∼50 to >80) is notable. These changes exceed the minimal clinically important difference (MCID) reported for the EQ-VAS in chronic conditions [57]. By contrast, lorazepam improved VAS modestly (50.80–59.20), suggesting that short-term benzodiazepine therapy may relieve sleep symptoms without fully restoring perceived health status. Herbal interventions such as chamomile tea, lavender oil inhalation and multi-herb formulas have been reported to improve both sleep and broader quality-of-life outcomes [58]. Likewise, studies on cannabinoid based medicines highlight improvements not only in insomnia symptoms, but also in anxiety, pain and overall life satisfaction [59,60]. This multidimensional effect may explain the greater gains observed in the herbal and cannabis groups.

Together, these results reinforce that insomnia management requires more than sleep induction and must also restore daily functioning and quality of life. Our findings suggest that culturally rooted herbal remedies and regulated cannabis oil preparations may offer a holistic benefit profile by targeting both nocturnal symptoms and daytime well-being. This is particularly relevant in thai and many african societies, where patients often prefer traditional or integrative approaches [22,61].

### 4.3 Safety and tolerability

Herbal and cannabis-based interventions demonstrated clinical improvements comparable to lorazepam, with adverse events that were mild and self-limiting. Although no serious adverse drug reactions were observed in any group, the present findings do not establish superior safety of integrative interventions over lorazepam. Rather, they suggest acceptable short term tolerability within the study duration [62]. Rebound insomnia was not systematically observed during the four-week intervention period. However, the short study duration precludes definitive conclusions regarding withdrawal related sleep disturbances. While benzodiazepines are well documented to cause rebound insomnia and dependence following discontinuation, evidence regarding similar phenomena with herbal or cannabis-based therapies remains limited and inconclusive. long-term, controlled studies are required to clarify safety, tolerance and withdrawal profiles [34,38]. This aligns with the literature emphasizing the gentler pharmacokinetic properties of botanicals [63].

The clinical improvements observed in the Suk-Sai-Yat group may be explained by multicomponent synergistic actions. Cannabidiol from *C.* *sativa* provides 5-HT1A mediated sedative and anxiolytic effects [16], while piperine-containing species (*Piper retrofractum, Piper nigrum*) offer anticonvulsant, muscle relaxant and anxiolytic properties [64]. Nutmeg (*Myristica fragrans*) has been shown to increase both light and deep sleep and ginger (*Zingiber officinale*) contributes to anxiolytic and neuroprotective effects [65]. Borneol from *Cinnamomum camphora* is a well-known sedative hypnotic agent [66]. Together, these botanicals may produce a balanced hypnotic profile with fewer adverse effects than single-compound hypnotics such as benzodiazepines.

## 5 Conclusions

This randomized controlled trial shows that Suk-Sai-Yat, a traditional Thai herbal remedy, and *Cannabis sativa* oil (Deja formula) significantly improve sleep quality and health related quality of life in patients with chronic insomnia. The clinical benefits of both integrative interventions were comparable to lorazepam over a four week treatment period. Both herbal and cannabis-based therapies were well tolerated, with only mild adverse events observed, supporting their suitability as non-benzodiazepine options in insomnia management. From an integrative medicine perspective, these findings highlight the value of traditional and botanical therapies as complementary or alternative approaches that address the multifactorial nature of insomnia and promote overall well-being.

Further studies are warranted to assess long-term outcomes and their potential role in reducing reliance on conventional hypnotic medications.

## CRediT authorship contribution statement

**Naruwat Pakdee:** Writing – review & editing, Methodology, Data curation, Conceptualization. **Nitcha Sribunrieng:** Writing – review & editing, Validation, Methodology, Formal analysis, Data curation. **Ronnachai Poowanna:** Writing – review & editing, Writing – original draft, Visualization, Validation, Methodology, Investigation, Formal analysis, Data curation.

## Limitations

This study has several limitations. First, the open-label design may have introduced performance bias, as participants and care providers were not blinded to treatment allocation due to the distinct characteristics of the interventions. Second, the relatively small sample size and short intervention period may limit the precision of the estimates and the ability to detect rare adverse events. Third, the study was conducted at a single center in a specific cultural and healthcare context, which may limit the generalizability of the findings to other populations or settings. In addition, the trial was not registered in a public clinical trial registry. Future multicenter studies with larger sample sizes, longer follow-up periods and trial registration are warranted to confirm and extend these findings.

## Institutional review board statement

The study protocol was reviewed and approved by the Human Research Ethics Subcommittee of Rajamangala University of Technology Isan Sakon Nakhon Campus, Phang Khon, Sakonnakhon 47160, Thailand (approval number: HEC-04-66-015). This trial complied with the principles of the Declaration of Helsinki. All participants were informed about the study objectives, procedures, potential risks, and benefits before providing written informed consent. Patient data confidentiality was maintained throughout the study.

## Data availability statement

The data that support the findings of this study are not publicly available due to privacy restrictions but are available from the corresponding author upon request.

## Funding

No.

## Declaration of competing interest

The authors declare that they have no known competing financial interests or personal relationships that could have appeared to influence the work reported in this paper.
