# Supplementary material for: Integrative therapies for chronic insomnia: A randomized controlled trial of a traditional Thai Herbal Remedy and Cannabis sativa oil
Source: Sleep Med X. 2026 Jan 17;11:100173. doi: 10.1016/j.sleepx.2026.100173 (PMC12856596; doi:10.1016/j.sleepx.2026.100173)
Supplement: Multimedia component 1 [file mmc1.docx]

**Supplementary Materials**

**Integrative Therapies for Chronic Insomnia: A Randomized Controlled Trial of a Traditional Thai Herbal Remedy and *Cannabis sativa* Oil**

**Naruwat Pakdee, Nitcha Sribunrieng, Ronnachai Poowanna***

**Supplementary Table S1.** Comparison of Pittsburgh Sleep Quality Index (PSQI) subcomponents among the three groups after the 4-week intervention

| PSQI Component  (Score 0-3) | Baseline Profile  (all groups) | Post-treatment | | | *P-value* |
| --- | --- | --- | --- | --- | --- |
|  |  | Suk-Sai-Yat | Deja Formula | Lorazepam |  |
| Subjective sleep quality | 2.10 ± 0.59 | 1.05 ± 0.60 | 0.85 ± 0.55 | 0.95 ± 0.65 | 0.582 |
| Sleep latency | 1.65 ± 1.09 | 1.10 ± 0.85 | 0.80 ± 0.70 | 0.90 ± 0.75 | 0.415 |
| Sleep duration | 2.00 ± 0.57 | 1.00 ± 0.65 | 0.75 ± 0.50 | 0.85 ± 0.60 | 0.389 |
| Sleep efficiency | 1.90 ± 1.03 | 0.80 ± 0.75 | 0.60 ± 0.55 | 0.70 ± 0.65 | 0.671 |
| Sleep disturbances | 1.36 ± 0.50 | 1.20 ± 0.45 | 1.10 ± 0.40 | 1.15 ± 0.50 | 0.812 |
| Use of sleeping meds | 1.94 ± 1.33 | 0.40 ± 0.50 | 0.35 ± 0.45 | 0.45 ± 0.55 | 0.925 |
| Daytime dysfunction | 1.95 ± 1.02 | 1.05 ± 0.70 | 0.65 ± 0.60 | 0.80 ± 0.75 | 0.115 |
| Total (Global Score) | 12.9 ± 0.85 | 6.60 ± 3.66 | 5.10 ± 1.77 | 5.80 ± 2.95 | 0.243 |

**Note:** Data are presented as mean ± SD.

*P-value* derived from one-way ANOVA comparing the three groups. A *P-value* > 0.05 indicates no significant difference between the treatments.
